# Unveiling viewpoints on national food environment policies in the Dutch newspaper discourse: an interpretative media content analysis

**DOI:** 10.1186/s12966-024-01625-3

**Published:** 2024-07-24

**Authors:** Nine M. S. Droog, Coosje S. Dijkstra, Naomi van Selm, Maartje P. Poelman, Joreintje D. Mackenbach

**Affiliations:** 1 Amsterdam UMC location Vrije Universiteit Amsterdam, Public and Occupational Health, Amsterdam, the Netherlands; 2 Amsterdam Public Health Research Institute, Amsterdam, the Netherlands; 3 Department of Health Sciences, Vrije Universiteit Amsterdam, Amsterdam, the Netherlands; 4 Chair Group Consumption and Healthy Lifestyles, Wageningen University & Research, Wageningen, the Netherlands; 5 Amsterdam UMC location Vrije Universiteit Amsterdam, Epidemiology and Data Science, Amsterdam, the Netherlands; 6 Upstream Team, http://www.upstreamteam.nl, Amsterdam, the Netherlands

**Keywords:** Prevention, Nutrition policy, Obesity, Media attention, Framing, Taxation, Labeling

## Abstract

**Background:**

National food environment policies can contribute to the reduction of diet-related non-communicable diseases. Yet, their implementation in the Netherlands remains low. It has been hypothesized that the media can play a pivotal role in inducing spikes in policy attention, thereby shaping political action. The aim of this study was to examine the discourse on food policies in Dutch newspaper articles between 2000–2022, by analyzing arguments used by various actors.

**Methods:**

A systematic search in Nexis Uni was used to identify newspaper articles that covered national-level Dutch food environment policies published in seven Dutch national newspapers between 2000–2022. Covered policies were classified into six domains including food composition, labeling, promotion, prices, provision and retail and into the four stages of the policy cycle; policy formulation, decision-making, implementation, and evaluation. A grey literature search was used to identify food policies implemented during 2000–2022. Descriptive statistics were used to summarize coverage of policies over time, policy type and policy stage. An interpretive content analysis was performed on a random subsample of the newspaper articles to determine the actors, viewpoints and arguments of the food policies.

**Results:**

We identified 896 relevant newspaper articles. The coverage of food policies in newspapers was initially low but peaked in 2018/2021/2022. Through grey literature search we identified 6 food policies which were implemented or adjusted between 2000–2022. The majority of the newspaper articles reported on food pricing policies and were discussed in the policy formulation stage. Academics (mainly supportive) were the most and food industry (mostly opposing) the least cited actors. Supportive arguments highlighted health consequences, health inequalities and collective responsibility, whereas opposing arguments focused on unwanted governmental interference and ineffectiveness of policies.

**Conclusions:**

Dutch newspaper articles covering food policies represented a variety of actors and arguments, with individual versus collective responsibility for food choices playing a central role in the arguments. These insights may serve as a basis for further research into why certain arguments are used and their effect on policy attention and implementation.

**Supplementary Information:**

The online version contains supplementary material available at 10.1186/s12966-024-01625-3.

## Background

Globally, non-communicable diseases (NCDs) are the leading cause of death [1]. An unhealthy diet is the largest risk factor for NCDs, surpassing smoking [2]. Regulatory and policy measures that target the unhealthy food environment such as a sugar-sweetened beverages (SBB) tax can contribute to the reduction of diet-related NCDs [3–7]. These national food environment policies (hereafter referred to as food policies) are strongly advised by globally recognized expert organizations such as the United Nations, the World Health Organization, and the Task Force on Fiscal Policy for Health [8–12]. Yet, there is a wide variation between countries in the extent to which such food policies are implemented, with the Netherlands ranked as the second lowest country compared to eleven European countries [13–15]. In 2018, the Dutch National Prevention Agreements was launched, where a range of actors agreed on measures to reduce overweight (and smoking and problematic alcohol use). In response to this launch, academics and the National Institute for Public Health and the Environment argued that with these agreements, targets to reduce overweight would not be met and that additional policies would be necessary [16, 17]. Currently, there are no unhealthy food taxes, national restrictions on marketing of unhealthy foods, no subsidies on fruit and vegetables and no regulations on food placement and promotion in the Netherlands, although the Nutri-Score label was adopted as a voluntary front-of-pack label since 2024..

The process of food policy adoption and implementation is complex and depends on several interacting factors, including the perceived severity of the health problem, generation and interpretation of scientific evidence for effectiveness of food policies, industry group activities, and the political and public attitude towards food policies [18–23]. While politicians have the power to implement and change food policies, they often favor the status quo [24]. Political stability predominates, with substantial policy changes only occasionally emerging when new ideas manage to break through [24, 25]. This phenomenon is also described in the punctuated equilibrium theory, which states that there is political stability most of the time, but sometimes there is a spike in policy changes [25].

One of the factors that can play an important role in inducing spikes in policy changes and thereby shape political action is the media [26–33]. Media outlets such as (online) newspapers, radio and television determine what and who gets a platform, and as such, influence which issues rise or fall on the public agenda. Media outlets, and especially actors using media, can select, emphasize or omit specific aspects of the topic to shape the understanding of the covered topic (i.e., framing). Consequently, the framing of an issue of food policy may influence the collective attitude in society towards this issue [34–38]. Media attention occurs during all stages of the policy cycle by describing suggested food policies (agenda-setting and policy formulation), expressing preferences (decision-making), reporting on implementation progress (implementation) and reporting on impact (evaluation) [33, 39].

What actors are represented in newspapers and which viewpoints and arguments they propagate may play an important role in understanding the framing of food policies in the media, but a limited number of studies have investigated this. In the case of the Soft Drinks Industry Levy in the UK, public health advocates – who were in favor of the policy—were most prominently represented [40]. Australian researchers found that although newspaper coverage of food policies was low, the majority of the food policy issues covered in newspapers were positively displayed in terms of public health [41]. Regarding the arguments in the newspaper articles, UK researchers identified various arguments, including appropriateness of regulation, (lack of) evidence, consequences of implementation, legal issues and social responsibility [40]. This latter argument is in line with Beauchamp (1976) and was also identified by a German study [42, 43]. German researchers found that in the case of the sugar tax, the focus in newspapers was mainly on societal responsibility which was centered around the debate of binding measures and voluntary solutions by the food industry [43]. In addition, Australian researchers observed that arguments that were in favor of food policies tended to be framed in terms of public health or society benefits, whereas dismissive arguments tended to be framed in economic, practical or ideological terms [44].

To understand the potential links between newspaper coverage and food policy adoption and implementation in the Netherlands, we first need to get a comprehensive overview of the coverage of different food policies, the policy cycle phase to which they correspond and actors' views and arguments regarding food policy issues in Dutch newspapers. We aimed to examine the discourse on food policies in Dutch newspaper articles between 2000–2022 by analyzing arguments used by various actors.

## Methods

### Study design

We conducted a systematic document analysis as part of a historical qualitative study design. We chose newsprint media in line with Cicchini et al. [41], as this is a consistent and substantial news source [41]. Additionally, in line with Cicchini et al. [41], we selected approximately 20 years to cover a relatively long historical period [41]. We followed the Standards for Reporting Qualitative Research (SRQR) and the checklist including a detailed description of our methods can be found in the Supplementary materials [45].

### Data collection

To identify relevant publications, we conducted a systematic search in Nexis Uni (LexisNexis) from inception up to December 31, 2022, in collaboration with a medical information specialist of the university library. We included all national newspapers in the Netherlands in 2022 (Algemeen Dagblad, Nederlands Dagblad, NRC (Handelsblad & in de ochtend), Reformatorisch Dagblad, de Telegraaf, Trouw, de Volkskrant) except for Manifest as this is a very small newspaper [46]. Het Financieele Dagblad was not available in Nexis Uni and therefore excluded.

Based on a literature study and discussion among the research team, we searched for terms related to national-level Dutch food policies. Examples of search terms are: sugar-sweetened beverages tax, food choice logo, healthy canteen, food retail, food promotion ban, food/meal composition, reduce sugar/saturated fat/trans-fat/salt(see Supplementary Table 1 for the complete search strategy).

Two assessors (NMSD and NS) independently screened titles and full texts of newspapers for eligibility. A 10% random sample of all identified newspaper articles was assessed by a third, independent assessor (JDM). We resolved differences in judgment through a consensus procedure. To be included, newspaper articles had to (a) be published in Dutch; (b) be published between the years 2000 and 2022; and (c) report on a (proposed) compulsory national-level Dutch food policy, thereby excluding self-regulatory or non-binding agreements.

We used the Dutch descriptions of food policy classifications from Djojosoeparto et al., [15], which was derived from Swinburn et al., [47], to categorize food policies into domains [15, 47]. These domains included food composition (e.g., reformulation policies to improve the nutrient profile), food labeling (e.g., legislating front-of-pack label), food promotion (e.g., marketing ban), food prices (e.g., sugar tax), food provision (e.g., nutritional standards for healthy school canteens) and food in retail (e.g., zoning policies for fast food) (see Supplementary Methods section including Supplementary Table 2) [15]. In line with Cicchini et. al. (2022), we excluded the domain of food trade and investments because of the focus on national policies, as the domain food trade and investments are part of global commerce [41]. We considered policies regarding all foods and beverages. We chose to include breastfeeding policies as research suggests that breastfeeding may protect against non-communicable diseases [48]. We excluded newspaper articles about alcohol as this is mostly not studied as food but as a different subgroup [49–51].

We used the policy cycle from Jann & Wegrich [39] to determine the specific stage within the cycle to which the identified food policy was associated [39]. The described policy stages are categorized as agenda-setting (e.g., problem recognition and issue selection), policy formulation (e.g., suggestion possible policies), decision-making (e.g., decision to implement (or abolish) policy), implementation (e.g., implementation / abolishment process), and evaluation (e.g., effect policy) (see Supplementary Methods section) [39]. As the agenda-setting phase is explained as problem recognition and issue selection without the formulation of a policy which could counter this issue, the stage agenda-setting could not be assessed. A grey literature search was done to retrieve implemented food policies in the Netherlands between 2000–2022. We started with the overview of governmental food policies from the Food-EPI project in the Netherlands [15]. To find additional implemented policies, different governmental websites were used as a starting point, such as wetten.overheid.nl, rijksoverheid.nl, belastingdienst.nl, and voedingscentrum.nl. Additionally, we performed unsystematic Internet searchers for further information about implemented food policies. Based on Hilton et al., [40] and discussions among the research team, we made a classification of actor groups [40]. These actors included academics, consumers, policymakers, actors working in public health/ environmental organizations and actors working in the food industry (see Supplementary Methods section and Supplementary Table 3). If no information regarding an actor was available, we coded this as 'unknown'. Identified individuals and/or organizations in newspaper articles were considered to be actors when direct quotes were supplied in the articles or when individuals and/or organizations were mentioned in relation to the policy and their viewpoint was considered verifiable. We categorized the viewpoints of actors as 'supportive', 'opposed' or 'neutral' towards the food policy based on the described arguments in the newspaper article.

### Data analysis

**Fig. 1:**
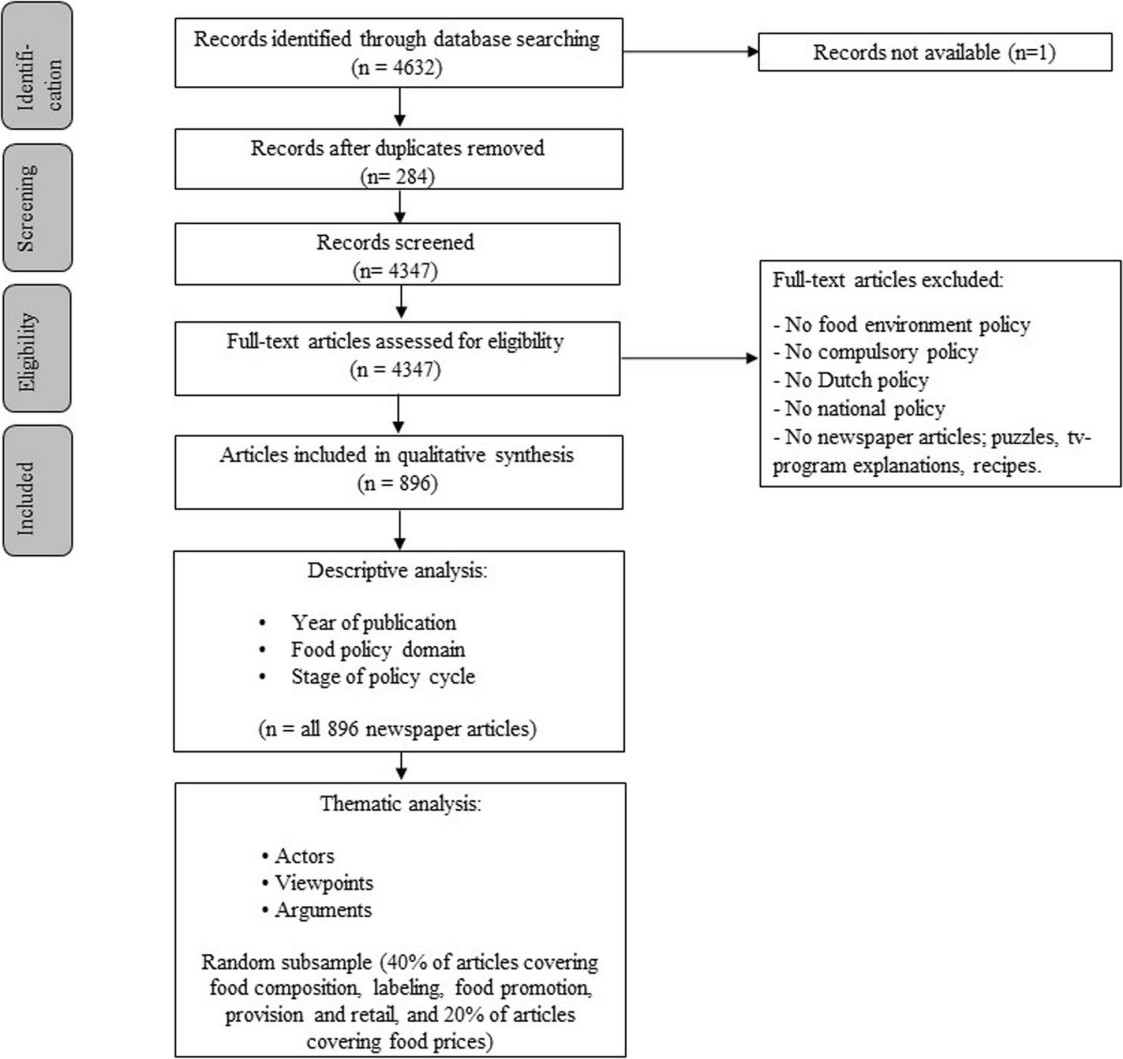
Flowchart of search and selection process, and overview of newspaper articles used for descriptive and thematic analysis, in study about actors’ viewpoints and arguments regarding national food policies in Dutch newspapers

One assessor (NMSD or NS) extracted details from all newspaper articles, including year of publication, covered food policy issues and the stage of the policy cycle, and these details were entered in Microsoft Excel for descriptive statistical purposes (Fig. 1).

From a random subsample, represented actors, viewpoints and arguments were identified (by ND) (Fig. 1). Randomly sorted newspaper articles were analyzed until no new or remarkable ratio of actors and represented arguments appeared, thus data saturation was reached. As newspaper articles could include multiple food policy issues, actors, viewpoints and arguments, newspaper articles were coded independently per food policy issue. Therefore, we coded until saturation was met per food policy domain. This resulted in coding of around a 20% random sample of the newspaper articles regarding food prices and around a 40% random sample of the newspaper articles covering other domains. We used a combination of deductive and inductive coding in the software program ATLAS.ti 23. We used deductive coding for the actors in the newspaper articles and the viewpoints and arguments of actors regarding the food policy issue through interpretive content analysis. In line with Rowbotham et al. [44] we initially deductively coded arguments into the themes of health, societal, economic, practical and cultural/ideological (see Supplementary Methods section and Supplementary Table 4) [44]. If arguments did not fit in these themes, we used inductive coding, which led to the formulation of additional themes based on the content analysis. If there was no clear argumentation, we labeled these articles as having ‘no argumentation’. Based on Hilton et al., [40],Rowbotham et al., [44] and discussion among the research team, we set up pilot arguments for each viewpoint and theme to clarify what type of arguments would fall under what themes (see Supplementary Methods section) [40, 44]. For instance, there were arguments regarding the need for government policies because it is the government’s responsibility to protect citizens (cultural/ideological argument) and regarding the need for government policies because they would have an effect on health that other solutions do not have (health argument). A 10% random sample of the analyzed subsample was coded by a second, independent assessor (NS). We resolved differences in judgment through a consensus procedure.

## Results

The literature search generated a total of 4632 references (see Fig. 1). After removing duplicates, 4347 references remained. In total, 896 articles satisfied the inclusion criteria. Articles were removed because no compulsory national-level Dutch food policy was covered or articles were not newspaper articles (e.g., puzzles, tv-program explanations and recipes.).

### Volume and content in Dutch newspapers

**Fig. 2:**
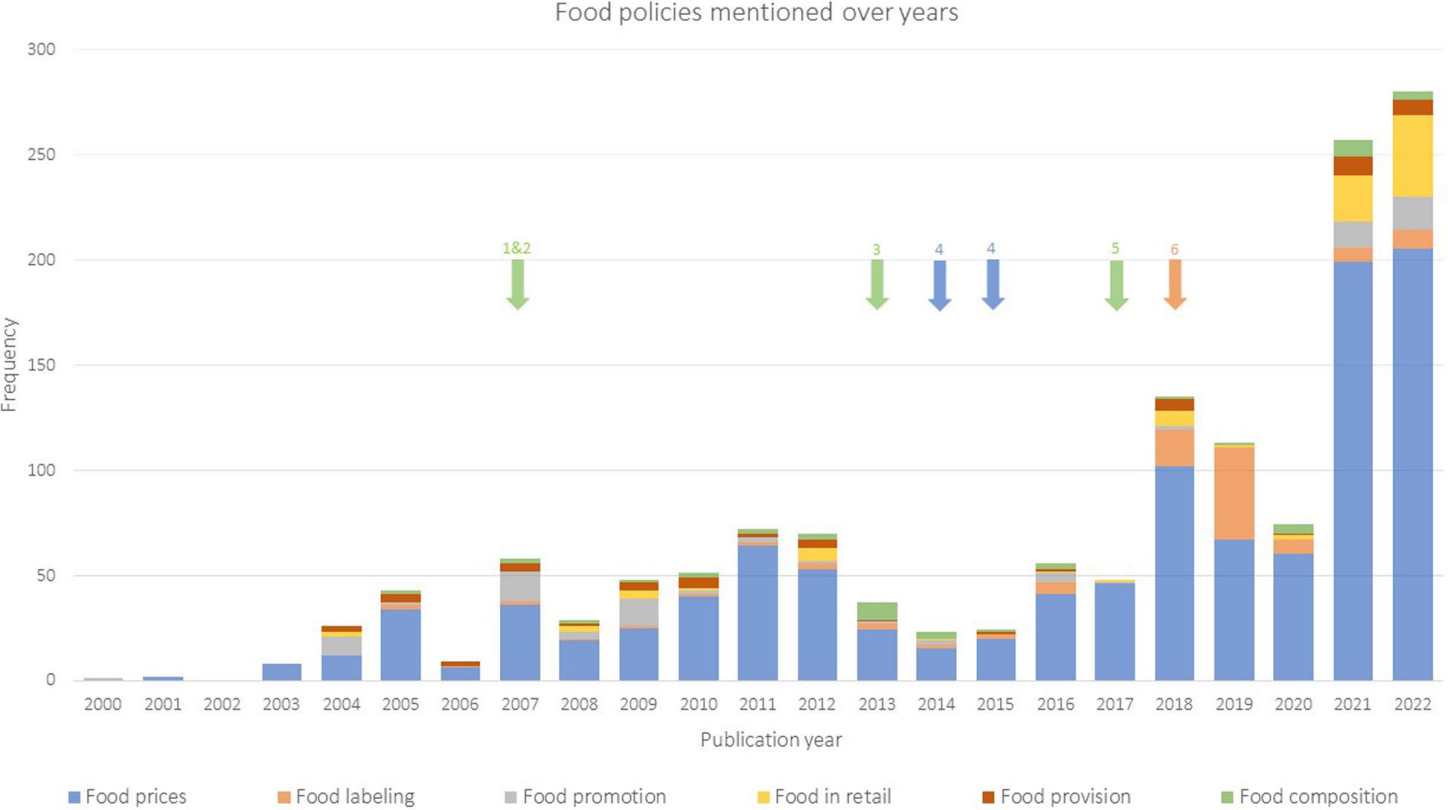
Frequency of national-level Dutch food policies cited in Dutch newspaper articles between 2000–2022. *Frequency is the number of times per year that a food policy was cited in the newspaper articles. *Arrows represent the timing of food policies implemented or adjusted in the Netherlands. Policy 1 = commodities act degree on four and bread; Policy 2 = commodities act degree on meat, minced meat and meat products; Policy 3 = maximum content of salt in bread; Policy 4 = law on consumption tax of alcohol-free drinks; Policy 5 = commodities act degree on preserved fruit products; Policy 6 = food choice logo

In the 896 included newspaper articles, food policies were mentioned 1464 times. The prevalence of food policy coverage in newspaper articles was low in the early 2000s and fluctuated until 2017 (Fig. 2). From 2018 on, the prevalence of food policy coverage increased, with peaks in 2018, 2021 and 2022. Regarding the content of newspaper articles, the majority (73,6%) of the newspaper articles reported on food price policies (Fig. 2). These articles mainly discussed meat, sugar, and fruit/vegetable taxation. Other food policy domains were mentioned less often (ranging from food composition (3,2%) to food labeling (7,5%)) (see Supplementary Table 5). The different food policy domains were roughly covered equally between newspapers (see Supplementary Fig. 1). Newspaper articles mainly described food policies in the stage of policy formulation (94,2%) (see Supplementary Table 6). Other stages of the policy cycle were discussed less frequently in the newspapers (ranging from implementation (0,1%) to decision-making (4,9%)). Through grey literature search we identified 6 food policies which were implemented or adjusted between 2000–2022 (arrows in Fig. 2).

- Policy 1: 2007: Commodities act degree on flour and bread: maximum salt content in bread reduced from 2.5% to 2.1% [52]
- Policy 2: 2007: Commodities act degree on meat, minced meat and meat products: maximum fat content in minced meat reduced from 35 to 25% [53]
- Policy 3: 2013: The maximum content of salt in bread went reduced from 2.1% to 1.8% [52].
- Policy 4: 2014 & 2015: Law on the consumption tax of alcohol-free drinks; 2014 the consumption tax went from €4,13 to €5,70 per hectoliter for fruit juice, vegetable juice, and mineral water. For lemonade the tax went from €5,50 to €7,79 per hectoliter. 2015; the consumption tax for all alcohol-free drinks went to €8,83 per hectoliter [54].
- Policy 5: 2017: Commodities act degree on preserved fruit products: minimal sugar content of jam reduced from 60 to 50% [55].
- Policy 6: 2018, The last ‘implemented’ policy was in fact an abolishment of an existing policy, namely the food choice logo ‘Vinkje’ [15].

The coverage of the six implemented policies in the newspapers was limited. Only the fat reduction in minced meat, the reduction of salt in bread and the abolishment of the food choice logo ‘Vinkje’ were mentioned in a few articles. The other implemented policies were not mentioned in the newspaper articles.

### Actors and their viewpoints

**Fig. 3:**
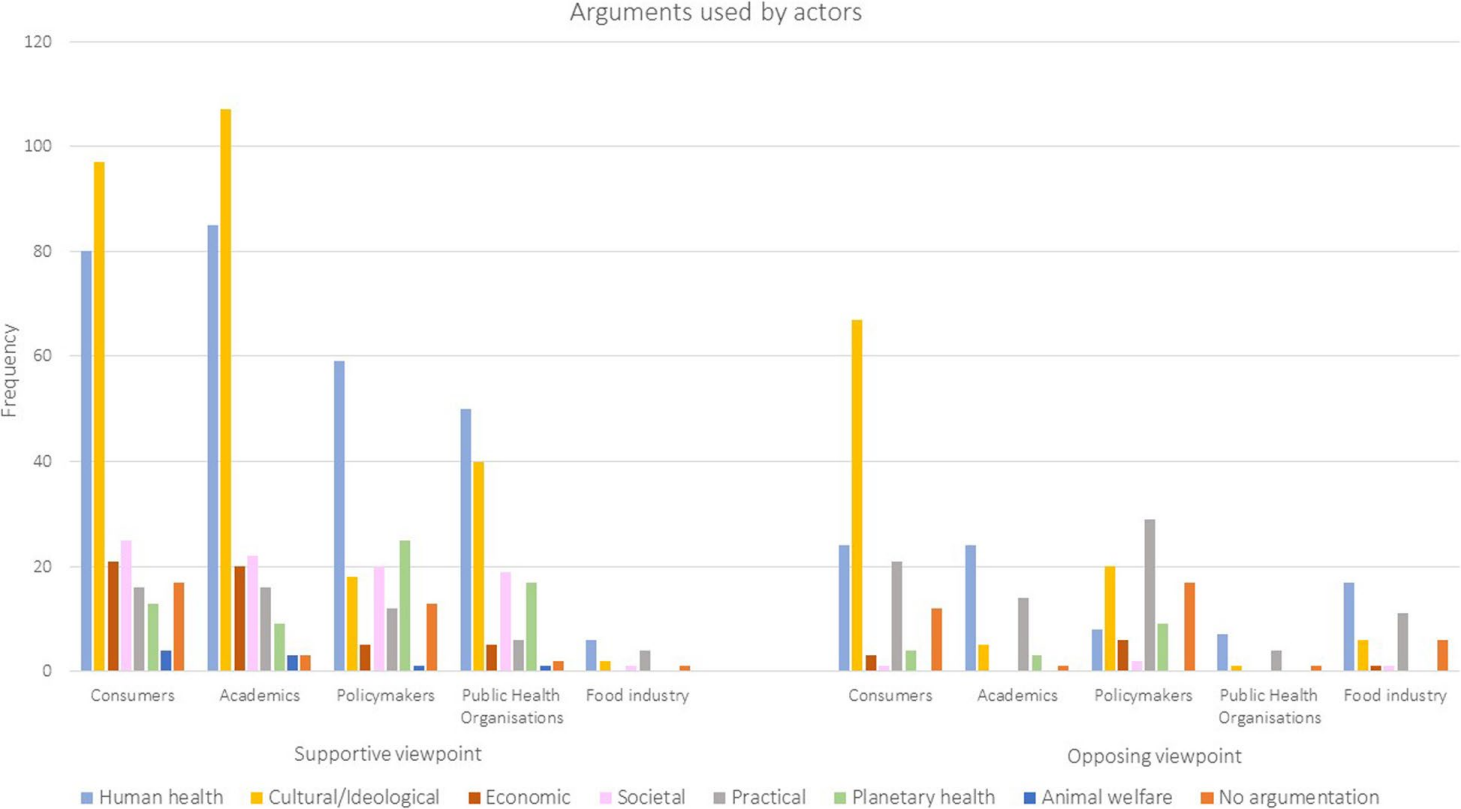
Frequency of themes of argumentation by actors regarding national-level Dutch food policies in Dutch newspaper articles between 2000–2022. Footnote: Frequency is the number of times per year that an argument was identified in the newspaper articles

**Fig. 4:**
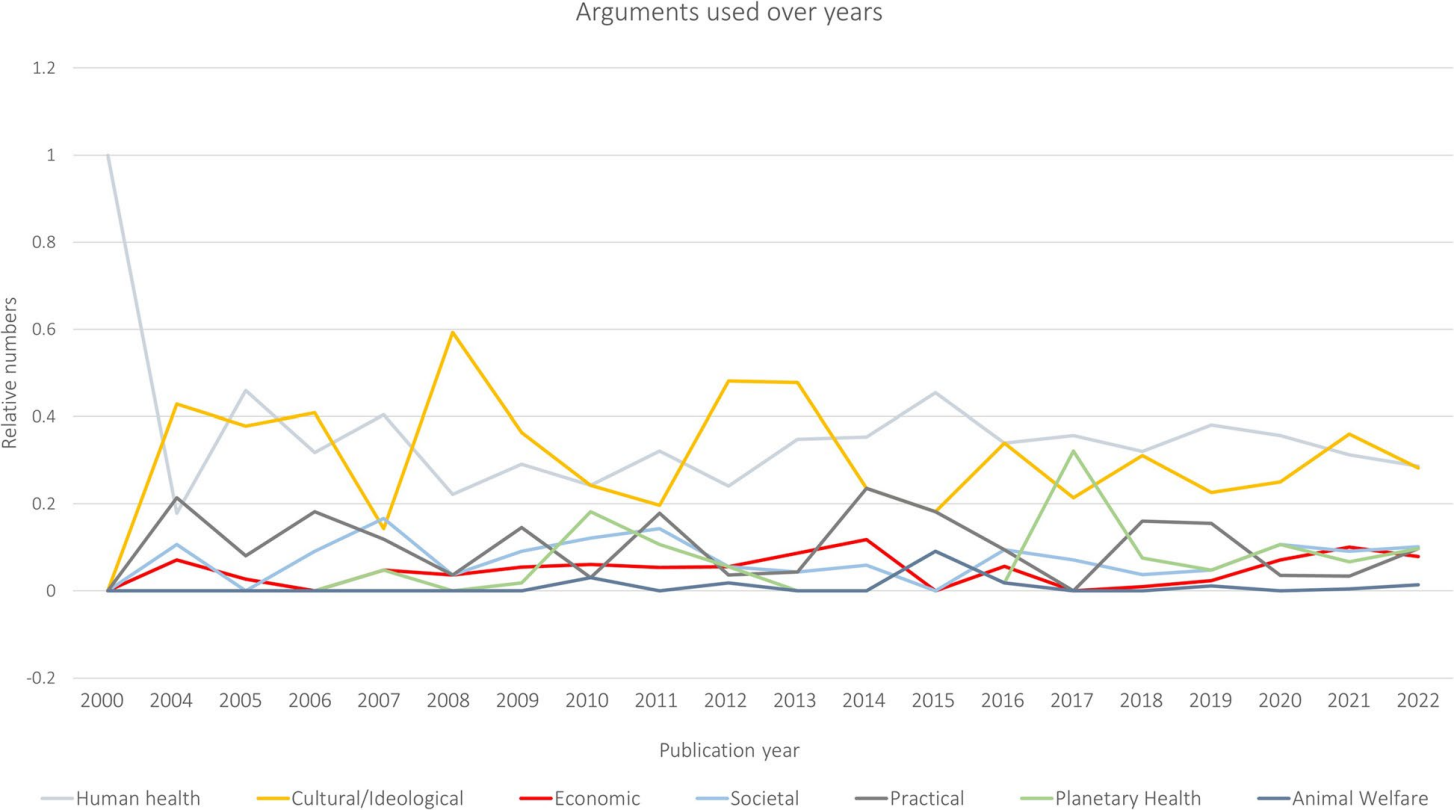
Relative contribution of themes of argumentation regarding national-level Dutch food policies in Dutch newspaper articles between 2000–2022. Footnote: Relative contribution is the proportion of arguments for a specific theme of all arguments identified during a year

In the random analyzed subsample of newspaper articles, consumers (34,6%) and academics (26,2%) were most frequently represented, followed by policymakers (21,6%), public health/environmental professionals (12,9%) and actors from the food industry (4,8%). A total of 1195 unique arguments were identified (see Supplementary Table 7). Consumers (66,1%), academics (84,7%), policymakers (59,3%) and public health/environmental professionals (90,9%) were mainly supportive of the food policies across all food domains (see Supplementary Table 8). Actors from the food industry (73,7%) predominantly opposed the food policies. The frequency of different themes used by actors can be found in Fig. 3. In general, a neutral viewpoint was rarely identified for all actors. There was no clear temporal pattern in representation of actors over time. Some arguments (e.g., human health, cultural/ideological) have been mentioned in newspapers since 2000, while others (e.g., planetary health, animal welfare) only emerged after 2010 (see Fig. 4). However, the ratio between supportive and opposing arguments did not change over time.

### Themes of arguments

In the random analyzed subsample of newspaper articles, we identified the themes of cultural/ideological (31.0%) and human health (30.8%) as the most prominent, followed by practical (11.4%), societal (7.8%) and economic (5.2%) themes, covering arguments in support of or opposing to the described food policies in the newspaper articles (see also Supplementary Table 9). In addition, we identified two new themes, including planetary health (6.8%) and animal welfare (0.8%). No argumentation was provided in 6.2% of the included newspaper articles.

#### Cultural/ideological

Cultural/ideological arguments were prevalent for both supporting and opposing the food policies of concern. Despite different food policies and actors, most identified arguments were very similar. Those in favor of the food policy (often academics and consumers) predominantly argued that current policies put too much emphasis on individual responsibility for food choices. Supporters argue that consumers, and especially children, do not truly have a ‘free choice’, and thus responsibility, in an obesogenic and unhealthy food environment (Quote 1–4, in Table 1). It was argued that governmental policies would ensure that responsibility for food choices was shifted more from the individual to a collective responsibility. These arguments were most frequently used for food retail and pricing policies. Opponents of the food policy (often policymakers, consumers and actors from the food industry) mainly argued generically that government interference is unwanted, or that the responsibility for food choices should lie with other entities such as individuals, parents, schools, food industry (Quote 8–10). Specifically for food promotion and provision policies, academics, consumers and public health professionals were often in favor of the proposed policies if they were aimed at children. However, if not aimed at children, arguments of consumers turned to unwanted government interference and displaced responsibility.

**Table 1:** Overview of supportive and opposing arguments towards national-level Dutch food policies identified in Dutch newspaper articles between 2000–2022

Themes of arguments	Viewpoint	Description	Quotes
**Cultural/Ideological**	**Supportive**	**Upstream action needed** • Policy is lacking (e.g. no policy in current situation) • Current policy is not sufficient (e.g. self-regulation) • The problem is too complex (e.g. obesity can only be solved by a higher upstream solution) • The food environment is the problem (e.g. people cannot be held accountable)	1. *“Interest groups have been advocating for legal agreements for years to force the food industry to improve their products.” – Public Health Organization (Nederlands Dagblad, 2021)* 2. *“But exactly this self-regulation has been strongly criticized by food organizations and nutritionists. Foodwatch states, “The sugar industry is pacifying the minister with commitments that hardly make citizens' healthier””- Public Health Organization (Telegraaf, 2016)* 3. *“Only with these measures there is a chance to stop the obesity epidemic.”—Academic (Trouw, 2009)* 4. *“The problem with the image of this "free" choice is that it is false. This is because today's consumers do not live in pure freedom, but in an environment that is constantly firing stimuli at them.” – Academic (Volkskrant, 2018)*
		**Government responsibility** • Duty of government to protect Dutch citizens or help them make the right choices	5. *“These rights dictate that the state must actively protect citizens, for example from the effects of overfeeding.”—Consumer (Trouw, 2021)*
		**True-pricing** • Products should have an ‘honest’ price, including the price for environmental pollution	6. *“Let's base taxes on the actual costs incurred for a given product and not fall into political reflexes. Let's judge ideas on their substance and necessity. A fair price for a fair piece of meat, pretty logical.”—Consumer (Trouw, 2016)*
		**Responsibility polluter** • The polluter must pay for their own environmental or health care costs	7. *“Greenhouse gas emissions, sugar consumption and meat production are a huge damage to the community. The 'polluter pays' principle is then only logical. It's just correct math.” – Food industry (Nederlands Dagblad, 2017)*
	**Opposing**	**’Nanny state’ argumentation** • Government should not interfere	8. *“No, no patronizing, replied previous Health Minister Edith Schippers (VVD) invariably to calls from health organizations and scientists to tackle excessive alcohol consumption, obesity and smoking. It is not up to The Hague (Parliament) to dictate to citizens what they eat or drink, Schippers believed.”- Policymaker (Trouw, 2018)*
		**Individual responsibility** • Behavior/ choices are individual/ parental responsibility	9. *“One respondent: “Don’t tell me what I eat or drink.””—Consumer (Telegraaf, 2022)*
		**Responsibility lies somewhere else** • Behavior/choices are the responsibility of the food industry/schools etc	10. *““The food industry must be addressed but they don't dare” writes one respondent. Another: “As soon as it comes to large food or drug manufacturers, the government suddenly doesn't give a damn or is unclear about rules and sanctions.””—Consumer (Telegraaf, 2018)*
		**Moral arguments** • Morally not justifiable to interfere	11. *“Too bad the way the RVZ proposes to intervene is morally unjustifiable.”—Academic (Volkskrant, 2011)*
		**Hidden agenda** • Government has different reasons for policy such as power, money or other stakes	12. *“Just a method for the government to rake in more money.”—Consumer (Telegraaf, 2021)*
**Human health**	**Supportive**	**Expected health impact** • Because there is a link between dietary intake/exposure and health (e.g. sugar, /fat and illness/ obesity) • Because the policy is effective in decreasing obesity/illness	13. *“Since the 1980s, the number of overweight people has increased from 27 to 43 percent. In the Netherlands, more than half of the people in their thirties and forties are overweight. That group has a higher risk of diabetes, cancer, cardiovascular disease and a whole bunch of other conditions. Poor eating habits are the second leading cause of early death in the Netherlands after smoking.”—Academic (Volkskrant, 2018)* 14. *“A ten percent increase in the price of soft drinks reduces consumption by an average of eight percent. That won't solve the obesity problem, but it's a start.”—Academic (NRC, 2013)*
	**Opposing**	**Expected health impact minimal** • Because the link between dietary intake/exposure and health is weak (e.g. fats are needed) • Because evidence for the effectiveness in decreasing obesity/illness is weak	15. *“Obesity is not due to advertisements, but to wrong choices.” – Food industry (Algemeen Dagblad, 2007)* 16. *“Norway and Sweden have long banned children's advertising of snacks and sweets on national channels, but the food producers say that Swedish children are still the fattest in Europe despite the ban.” – Food industry (Volkskrant, 2007)*
**Practical**	**Supportive**	**Expected impact (general)** • General positive impact of policy (e.g. excise taxes are effective) • Evidence that general policy is effective from previous examples (e.g. seat belt/smoking/alcohol)	17. *“It is generally known that the idea of true pricing does play a role in whether or not to purchase a product.”—Academic (Nederlands Dagblad, 2022)* 18. *“He refers to compulsory seat belts, setting speed limits, and excise taxes on cigarettes and alcohol. Superficially, these deprive us of freedom, but ultimately they provide a safer environment in which people can move more freely.”—Academic (Volkskrant, 2018)*
		**Conditional support** • Under the condition of changing practical issues of the policy, proof of effectiveness or if another policy is also implemented	19. *“Unhealthy food should only get more expensive if healthy food gets cheaper.”—Consumer (Telegraaf, 2007)*
		**Policy as leverage point** • The policy is used to force self-regulation (e.g. if the sugar level is not going down, we implement the policy)	20. *“Fast food restaurants must first be given the chance to make their menus healthier. But if they do not succeed, a minimum age can be used as an incentive.”—Academic (Trouw, 2022)*
	**Opposed**	**Expected impact minimal (general)** • Minimizing positive impact of policy by practical issues (e.g. flaws in policy, confusion)	21. *“The Dutch consumer union found the green and blue Checkmark “unclear and misleading.”” – Consumer (Reformatorisch Dagblad, 2019)*
		**Necessary support towards policy lacking** • Policy cannot be implemented because there is a lack of public or political will	22. *“But the proposal caused so much protest in the House of Representatives that Minister Staghouwer (agriculture) said that he does want a rigorous price increase.”—Policymaker (Trouw, 2022)*
		**Policy is not feasible** • Difficulties in implementation because of national or EU legislation • The policy is too complicated (IT or categorization difficulties)	23. *“According to Waalkens, the World Trade Organization or Brussels will never agree to the proposed meat tax of 85 cents per kilo of meat.”—Policymaker (Trouw, 2007)* 24. *“Prime Minister Rutte recently said in the House of Representatives that it is extremely complicated to determine whether a pizza or a jar of pasta sauce would be included [in the definition of fruit and vegetables]. There are also doubts whether the Tax Administration's computer systems can handle such a major change.”—Policymaker (Algemeen Dagblad, 2022)*
		**Policy is unnecessary** • Self-regulation is sufficient or effective	25. *“The major food manufacturer Unilever thinks that the government is not needed to make healthier products. “We have been working on new recipes and innovations for ages, without compromising on taste.” – Food industry (Telegraaf, 2016)*
**Societal**	**Supportive**	**Policy protects vulnerable people** e.g. children, elderly, low-income • Protecting vulnerable people from obesity/illness, overconsumption, food temptations, abundant food supply and triggers	26. *“In recent years, the health disparities between the highly educated and less educated have been widening. It is known that lower educated people eat unhealthier, drink more and smoke more often.” – Public Health Organization (NRC, 2010)*
	Opposing	**Policy will harm vulnerable people** e.g. children, elderly, low-income • Harms vulnerable people by enlarging inequalities (e.g. harming low-income groups by taxation)	27. *“Lower income groups may be disadvantaged by a sugar tax.”—Academic (NRC, 2016)*
**Planetary health**	**Supportive**	**Expected planetary impact** • Link between dietary intake/exposure and climate, sustainability and biodiversity (e.g. meat and climate) • (Evidence of) Effective policy for climate, sustainability and biodiversity	28. *“Residents of the world's most livestock-dense country (yes, the Netherlands) should consume significantly less animal protein and significantly more plant protein because raising animals for human consumption puts a huge strain on natural resources.”—Policymaker (NRC, 2010)* 29. *“International research shows that making meat more expensive affects meat consumption.”—Academic (Volkskrant, 2012)*
	**Opposing**	**Expected planetary impact** • Low policy expectations for effect on climate, sustainability and biodiversity	30. *“But meat is already a bargain so it is not expected that a modest price increase will result in eating less meat by the consumer.”—Consumer (Trouw, 2021)*
**No argumentation**	**Supportive**		31. *“The PvdA (Labour Party) supports the proposal.” – Policymaker (Algemeen Dagblad, 2006)*
	**Opposing**		32. *“Koninklijke Horeca Nederland (KHN) is not interested in a ban”. – Food industry (Nederlands Dagblad, 2009)*
**Economic**	**Supportive**	**Expected economic impact** • Raises revenues (which can be used for different reasons) • Sketching the current situation of obesity/ illness and rising healthcare costs	33. *“A poll revealed that 63 percent of Dutch people agree with “a fair and true meat price” if the revenues are used to offset the purchasing power of lower incomes, make fruits and vegetables cheaper and give farmers sustainability support.”—Consumer (Volkskrant, 2022)* 34. *“By doing so, we can prevent that soon we will all have to pay to afford the costs of all those sick people.”- Academic (Algemeen Dagblad, 2008)*
	**Opposed**	**Expected economic impact minimal** • Retailers get extra costs • Retailers lose retail market/ move abroad	35. *“The reduction in meat consumption that is supposed to result from the measure will affect exactly those farmers who take care of their animals and who, as a result, have to charge a bit more for their meat anyway. That meat will then definitely become too expensive.”—Consumer (NRC, 2011)* 36. *“If only the Netherlands starts taxing more, it could lead to more exports or relocation of production, according to the climate committee Van Geest.”—Policymaker (Telegraaf, 2021)*
**Animal welfare**	**Supportive**	**Expected animal welfare impact** • Effective policy for animal welfare	37. *“The Pigs in Distress Foundation (Stichting Varkens in Nood) advocates for excising taxes on meat. The organization argues that with that money, the ailing pig sector can be reorganized.” – Public Health Organization (Algemeen Dagblad, 2015)*
	**Opposing**	**-**	*-*

#### Human health

Human health arguments were also often prevalent for both supporting and opposing viewpoints regarding food policies. However, the identified arguments focused on different aspects of health. Supporters (most often academics and consumers) highlighted the current overconsumption of unhealthy foods (e.g., high in sugar, saturated fat or red/processed meat), and its linkage to possible negative health consequences (e.g., high prevalence of and increased risks for overweight, obesity, non-communicable diseases) (Quote 13). Furthermore, the supportive arguments also described the effectiveness, along with supporting evidence, of policies (in)directly decreasing obesity and negative health consequences (Quote 14). However, academics questioned the positive impact of food labeling policies on health outcomes (Quote 16). Although actors from the food industry mostly emphasized the lack of evidence regarding the impact of food policies on health, in the case of food labeling policies they argued the potential positive effect on health. Yet, food industry actors generally opposed a ban on food promotion as they argued a lack of positive impact on health.

#### Practical

Practical arguments included arguments such as the expected general impact and feasibility of the proposed food policy. In general, opposing arguments tended to be centered around practical issues of the development/implementation of the food policy, which were very specific per food policy. Academics and consumers argued a minimal anticipated impact of food labeling policies, citing flaws of the policy or pointing to past (unsuccessful) attempts as evidence (Quote 21). Furthermore, the feasibility of food pricing policies through European legislation or IT problems were often brought up by policymakers (Quote 23,24). In addition, actors from the food industry were prominent in arguing that the food promotion policies were practically not necessary because self-regulation (of the food industry) was sufficient (Quote 25). Exceptions were supportive arguments highlighting the effectiveness of government policies on health in general, which was mostly done by academics and consumers (Quote 17,18).

#### Societal

Societal arguments predominantly highlighted the anticipated positive impact of the proposed food policy on vulnerable people. However, these vulnerable groups differed per food domain. Societal arguments were mostly used for food pricing, promotion and retail policies. Academics, consumers and policymakers highlighted the need for food retail policies as the health of children would be at stake. Academics and consumers also argued the need for food promotion policies, however specific arguments from policymakers were not identified. For food pricing policies, academics, consumers and policymakers mainly highlighted the positive impact on the health of individuals with a lower socioeconomic position (Quote 26). Occasionally policymakers used ‘widening health inequalities’ as an argument opposing pricing policies (Quote 27).

#### Planetary health

Planetary health arguments highlighted the expected impact on the planet by the proposed food policy, which was almost exclusively used for arguments supporting a meat tax. Policymakers and environmental organizations’ professionals mostly highlighted the current global or national situation regarding climate, sustainability and/or biodiversity or focused on the effectiveness (and evidence) of the policy by (in)directly contributing to a positive impact on the planet (Quote 28,29).

#### Economic

Economic arguments predominantly highlighted the expected positive economic impact of the proposed food policy. Academics often stated the current situation regarding rising health care costs (that would decline due to the policies) or mentioned that possible revenues of particular food policies (e.g., sugar tax) could be used to lower the prices of healthy foods like fruit/vegetables (Quote 33,34). However, policymakers mainly discussed adverse economic consequences, particularly resulting from price policies, emphasizing issues as inflation and negative impacts on the retail market (Quote 35,36).

#### Animal welfare

Animal welfare arguments solely highlighted the positive expected impact on animal welfare by the proposed food policy, which was only used in the context of the meat tax (Quote 37). This was mainly done by academics and consumers. Newspaper articles never exclusively focused on animal welfare arguments but always in combination with arguments around planetary health.

## Discussion

We demonstrated that between 2000–2017 the coverage of food policies in Dutch newspapers was relatively low, but increased from 2018. The majority of the newspaper articles reporting on food policies were regarding food prices and described in the stage of policy formulation. Consumers’ and academics’ viewpoints were most often reflected and they were mostly supportive of the food policies. Viewpoints from actors working in the food industry actors were least reflected and mostly opposed the food policies. Arguments in favor of food policies tended to be centered around human health (e.g., prevention), ideological (e.g., collective responsibility) or societal (e.g., reducing health inequalities) arguments, whereas opposing arguments tended to be centered around ideological (e.g., paternalism), practical (e.g., infeasibility) or human health (e.g., ineffectiveness) arguments.

The temporal patterns of food policy coverage in newspaper articles are difficult to compare, as the only comparable study in Australia shows a different pattern. Therefore, these patterns are likely to be context-dependent. The Australian study showed a pattern with peaks in 2006, 2011 and 2017–2019 [41]. In the Netherlands, the steep increase in food policy coverage around 2018 may be linked to the Dutch National Prevention Agreement that was launched in 2018 [56], which also explains why most articles focus on the policy formulation stage. In this agreement, a range of actors agreed on measures to reduce overweight (and smoking and problematic alcohol use). In response to this launch, academics and the National Institute for Public Health and the Environment argued that with these agreements, targets to reduce overweight would not be met and that additional policies would be necessary [16, 17]. This may have pushed these actors to seek press attention for additional policies such as a sugar tax. The two new themes identified as compared to an Australian study [44], planetary health and animal welfare, may also be attributable to increased awareness of global warming in the past years.

Our observations that academics were the most and food industry actors the least cited actors in newspaper articles are consistent with findings from the UK [40]. Firstly, given the low rates of food policy implementation in the Netherlands, those supporting food policy implementation are more inclined to seek media attention than those opposing food policy implementation because no media attention is likely supportive of maintaining the status quo. However, it could also be that journalists more often seek for academics’ views. Also, food industry actors may have deliberately opted to not be represented in newspaper articles as a tactical strategy to maintain a low profile in the public discourse since they may have other, less visible lobbying strategies to influence policy processes [57]. Academics may have less ability to voice their viewpoint through such quiet lobbying strategies, and newspaper media coverage is then an important strategy to share scientific insights and exercise their discursive power to reach the public.

However, not only frequency of representation but also arguments used may be indicative of actors’ discursive power. In line with Beauchamp (1976), we found that individual versus collective responsibility for food choices played an important role in Dutch newspaper articles [42]. This was also identified in a German study regarding newspaper coverage of the sugar tax [43]. Supporters argued that current policies put too much emphasis on individual responsibility, while individuals (consumers and especially children) do not truly have a ‘free choice’ and thus responsibility in an unhealthy and obesogenic food environment. Opponents argued that the government should not interfere in individual choices, thus deeming most food policies inappropriate and unjustifiable. Previous evidence suggests that unhealthy commodity industries (e.g., ultra processed food) have effectively pushed the narrative around diet and food choices towards individual responsibility [58]. As Michielsen (2022) argued around the topic of meat consumption [59], it appears that opponents tend to be more disapproving of government interference in general rather than of food specific policies. Indeed, supporters made use of substantive ideological arguments regarding food policies whereas for opponents these arguments were lacking. Furthermore, we found that opposing arguments relied on either the ineffectiveness of governmental policies to curb the health problem or the infeasibility of implementing the food policy. This is in line The Corporate Political Activity Model which highlights different strategies from unhealthy commodity industries to stop governments and global organizations from adopting effective public health policies [60]. For instance in an UK study, researchers also found that unhealthy commodity industries aimed to influence the view of the public and politicians regarding health issues by propagating arguments involving the complexity of these issues, indicating ineffectiveness of governmental policies [61]. We observed that where supporters tended to focus on the health problems/consequences, opponents rarely minimized these health problems/consequences, but focused on questions around the effectiveness of policies on these health problems/consequences. If in legislative arenas arguments of supporters and opponents also misalign, this may partly account for the lack of progress in the implementation of food policies.

In this study, we found that if economic arguments were used, these were almost exclusively in favor of implementation of food policies. In contrast, a UK study found economic arguments to be used both for supporting and opposing policies [44]. One explanation may be that this UK study included newspaper articles up until 2016. Since then, real-world evidence has countered a number of economic arguments such as the loss of jobs and profits following food policy implementation [62–64]. For instance, the Chilean food policy package did not have a negative impact on labor outcomes [62].

### Strengths and limitations

Strengths of this study were the 22-year timeframe, detailed content analysis, coverage of volume and content of newspapers as well as various food policies, actors and arguments. This study also has limitations. Despite our extensive search strategy, we may have missed important search terms, e.g., those used during the early 2000s. Furthermore, we excluded food trade, food investment and self-regulation of the food industry which could have led to different viewpoints and arguments. In addition, we chose newspaper media, in line with previous research, as this has been a consistent and substantial media outlet for the past two decades. Yet, by exclusively focusing on newspaper articles a selection of arguments and actors was analyzed. Lastly, by grouping actors into main categories, the general result could have lost nuance as within actor groups different viewpoints and interests can be held.

### Implications for practice and research

The findings from this study have implications for practice and research. Firstly, for practice, our results provide public health advocates with a clear overview of arguments used by opponents that they can try to refute. For instance, the rejection of government interference in general may be counteracted through arguments around the legal responsibility of governments to protect their citizens from harmful influences [65–67]. As UK researchers suggested, public health advocates could shift their focus articulating policies towards ‘empowerment of the public’ rather than propagating arguments around restricting certain consumer behaviours [68]. The findings from this study also have implications for future research. As the punctuated equilibrium theory describes, most of the time there is political stability, but sometimes there is a spike in policy changes [25]. One of the factors that can play an important role in inducing spikes in policy changes and thereby potentially shape political action is the media. Future research should determine if the increasing newspaper coverage, mainly of policies in a policy formulation stage, is an indicator for increased food policy adoption and implementation. One potential analytical approach for this could be the Granger causality test that investigates whether time series in the exposure can correctly predict time series in the outcome [69]. Secondly, future research could explore how actors’ viewpoints and arguments change over time and between types of newspapers. This could provide a further understanding of when and why certain arguments are abandoned over time, and which arguments remain as they may considered effective. Finally, we were unable to identify the quality of argumentation, i.e., distinguishing between facts, opinions and myths, as there is no valid methodology yet to do so. Considering the rising infodemic, more attention should be paid to the distinction between the quality of arguments regarding the type of actors (70).

## Conclusions

As the framing of public health issues in media potentially influences public opinion and policy adoption and implementation, we aimed to get more insight into the actors’ viewpoints and arguments in Dutch newspapers regarding food policies. This study found that arguments tended to concentrate on roles of responsibility whereby opponents tended to reject government interference in general rather than of food specific policies. Insights from this study may serve as a basis for further and deepening research into why certain arguments are used and what their effect is on collective attitudes and policy action.

### Supplementary Information

Supplementary Material 1.

Supplementary Material 2.

## Data Availability

The datasets generated and/or analyzed during the current study are available through Supplementary Materials.

## Abbreviations

NCDs: Non-communicable diseases
SSB: Sugar-sweetened beverages
SRQR: Standards for Reporting Qualitative Research
