# Supplementary material for: Unveiling viewpoints on national food environment policies in the Dutch newspaper discourse: an interpretative media content analysis
Source: Int J Behav Nutr Phys Act. 2024 Jul 24;21:80. doi: 10.1186/s12966-024-01625-3 (PMC11267762; doi:10.1186/s12966-024-01625-3)
Supplement: Supplementary file 1 — Supplementary Material 1. [file 12966_2024_1625_MOESM1_ESM.docx]

**Supplementary materials**Unveiling viewpoints on national food environment policies in the Dutch newspaper discourse: an interpretative media content analysis

*Nine M.S. Droog, Coosje S. Dijkstra, Naomi van Selm, Maartje P. Poelman, Joreintje D. Mackenbach*

**Contents**

[Extended methods 1](#_Toc167881834)

[Study Design 2](#_Toc167881835)

[Qualitative approach and paradigm 2](#_Toc167881836)

[Data Collection 2](#_Toc167881837)

[Complete search strategy Nexis Uni 2](#_Toc167881838)

[Classification of food policies from Djojosoeparto et al., (2022) 3](#_Toc167881839)

[Classification of policy cycle stages 7](#_Toc167881840)

[Classification of actors 8](#_Toc167881841)

[Classification of themes of argumentation 10](#_Toc167881842)

[Data analysis 10](#_Toc167881843)

[Pilot arguments 10](#_Toc167881844)

[Reflexivity 12](#_Toc167881845)

[Extended results 14](#_Toc167881846)

[Search and selection process 14](#_Toc167881847)

[Overview of volume and content of food policies in Dutch newspapers 14](#_Toc167881848)

[Overview of policy cycle stages 16](#_Toc167881849)

[Overview of viewpoint actors and arguments 17](#_Toc167881850)

[SRQR Checklist 20](#_Toc167881851)

[References 24](#_Toc167881852)

# **Extended methods**

In this section, we provide additional details on the methods employed for this study.

## Study Design

### Qualitative approach and paradigm

We conducted a systematic document analysis as part of a historical qualitative study design. As we looked at viewpoints and arguments from different actors, and their perception/reality regarding the food policies, we used a constructivist approach. In addition, we applied a combination of an inductive and deductive approach. As arguments for national-level food policies can overlap from different countries we started with pre-identified themes and arguments. However, as there was no literature from the Netherlands, and arguments per country could differ, we combined this with an inductive approach.

## Data Collection

### Complete search strategy Nexis Uni

To identify the relevant publications, we conducted a systematic search in Nexis Uni (LexisNexis) from inception up to December 31, 2022, in collaboration with a medical information specialist of the university library. Nexis Uni is an online database that includes full newspaper articles from national and regional newspapers. We included all national newspapers in the Netherlands in 2022 (Algemeen Dagblad, Nederlands Dagblad, NRC (Handelsblad & in de ochtend), Reformatorisch Dagblad, de Telegraaf, Trouw, de Volkskrant) except for Manifest as this is a very small newspaper (1). Het Financieele Dagblad was not available in Nexis Uni, and therefore we could not include this newspaper in the search. We based search terms on literature study and discussion amongst the research team (Table 1).

*Supplementary Table 1: Nexis Uni (LexisNexis) Session Results (30 March 2023) of the study about actors’ viewpoints and arguments regarding food policies in Dutch newspapers.*

| Search | Query | Items found |
| --- | --- | --- |
| #2 | Limits:  AD Algemeen Dagblad / De Telegraaf / De Volkskrant / Nederlands Dagblad / NRC (NRC in de ochtend + NRC Handelsblad) / Reformatorisch Dagblad / Trouw | 4,632 |
| #1 | (suikertaks OR "suiker-taks" OR suikertax OR "suiker-tax" OR (belasting w/3 suiker*) OR (belasting w/3 "ongezonde voeding*") OR voedingsaanbod OR (voeding w/3 aanbod) OR fortificatie OR "nutri-score*" OR "voedingskeuze Logo" OR "voedselkeuze Logo" OR "voedingskeuzelogo" OR "voedselkeuzelogo" OR reclameverbod* OR productpromotie OR "product promotie" OR "Akkoord verbetering maaltijdsamenstelling" OR "Akkoord verbetering productsamenstelling" OR voedingsrichtlijn* OR schoolmaaltijd* OR (gezond* w/3 leefomgeving) OR (gezond* w/3 kantine*) OR (subsidie w/3 "gezonde voeding") OR (sponsoring w/3 "ongezonde voeding*") OR "true pricing" OR (transvet* w/3 (verbod* OR verbied*)) OR (btw w/3 "fruit en groente") OR (verlag* w/3 " fruit en groente") OR (aanb* w/3 "gezonde voeding*") OR (accijns w/3 voeding*) OR (verminder* w/3 zout*) OR (verminder* w/3 verzadigd*) OR (verminder* w/3 suiker*) OR (verminder* w/3 transvet*) OR productverbetering* OR "product-verbetering*" OR (voeding w/3 sportkantine*) OR etikettering OR voedselnorm* OR verkoopautoma* OR voedselmarketing OR "voedsel-marketing" OR (voedsel w/3 belasting*) OR voedselbelasting* OR (voedsel w/3 retail*) OR voedselverkoop OR (voedsel w/3 verkoop) OR vleestaks OR "vlees-taks" OR vleestax OR "vlees-tax" OR (promot* w/3 "gezonde voeding*") OR voedselaanbod OR (voedsel w/3 aanbod) OR fastfoodstop OR "fastfood stop" OR "fast food stop" OR snacktaks OR "snack-taks" OR snacktax OR "snack-tax" OR vettaks OR "vet-taks" OR vettax OR "vet-tax") | 10,000+ |

### Classification of food policies from Djojosoeparto et al., (2022)

We used the Dutch descriptions of food policy classifications from Djojosoeparto et al., (2022), which was derived from Swinburn et al., (2013), to categorize food policies into domains (Table 2) (2,3). These domains included food composition, food labeling, food promotion, food prices, food provision and food in retail. The domain of food composition encompassed measures for processed foods such as fortification or restrictions of nutritional additions. The domain of food labeling included measures for packages such as nutritional information and food choice logos. The domain of food promotion included measures regarding the exposure and power of food marketing of unhealthy foods for instance a marketing ban. The domain of food prices encompassed price measures such as taxation on sugar and subsidies on fruit and vegetables. The domain of food provision included healthy food services policies in government-funded settings for example ‘healthy canteens’. The domain of food in retail included measures to influence the availability of foods in neighborhoods such as outlet density, location and product placement. In addition, we added breastfeeding policies as research suggests that breastfeeding may protect against non-communicable diseases (4). We excluded newspaper articles about alcohol as this is mostly not studied as food but as a different subgroup (5-7).

*Supplementary Table 2: Classification of food policies directly copied from Djojosoeparto et al., (2022).*

| Food-EPI Domain | Food-EPI Indicators |
| --- | --- |
| Domain 1 – Food composition  Food composition targets/standards/restrictions for processed foods: There are government systems implemented to ensure that, where practicable, processed foods minimize the energy density and the nutrients of concern (salt, saturated fat, trans fat, added sugar). | COMP1 Food composition targets/standards/restrictions have been established by the government for the content of the nutrients of concern (trans fats, added sugars, salt, saturated fat) in industrially processed foods, in particular for those food groups that are major contributors to population intakes of those nutrients of concern. |
|  | COMP2 Food composition targets/standards/restrictions have been established by the government for the content of the nutrients of concern (trans fats, added sugars, salt, saturated fat) in meals sold from food service outlets, in particular for those food groups that are major contributors to population intakes of those nutrients of concern. |
| Domain 2 – Food labeling  There is a regulatory system implemented by the government for consumer-oriented labeling on food packaging and menu boards in restaurants to enable consumers to easily make informed food choices and to prevent misleading claims. | LABEL1 Ingredient lists and nutrient declarations in line with Codex recommendations are present on the labels of all packaged foods. |
|  | LABEL2 Evidence-based regulations are in place for approving and/or reviewing claims on foods, so that consumers are protected against unsubstantiated and misleading nutrition and health claims. |
|  | LABEL3 Evidence-based regulations are in place for approving and/or reviewing claims on foods, so that consumers are protected against unsubstantiated and misleading nutrition and health claims. |
|  | LABEL4 A simple and clearly-visible system of labeling the menu boards of all quick service restaurants (i.e. fast food chains) is applied by the government, which allows consumers to interpret the nutrient quality and energy content of foods and meals on sale. |
| Domain 3 – Food promotion  There is a comprehensive policy implemented by the government to reduce the impact (exposure and power) of promotion of unhealthy foods to children across all media.  • Exposure of food marketing concerns the reach and frequency of a marketing message. This is dependent upon the media or channels which are used to market foods.  • The power of food marketing concerns the creative content of the marketing message. For example, using cartoons or celebrities enhances the power (or persuasiveness) of a marketing message because such strategies are attractive to children. | PROMO1 Effective policies are implemented by the government to restrict exposure and power of promotion of unhealthy foods to children including adolescents through broadcast media (TV, radio). |
|  | PROMO2 Effective policies are implemented by the government to restrict exposure and power of promotion of unhealthy foods to children including adolescents through online and social media. |
|  | PROMO3 Effective policies are implemented by the government to restrict exposure and power of promotion of unhealthy foods to children including adolescents through nonbroadcast media other than packaging and online/social media. |
|  | PROMO4 Effective policies are implemented by the government to ensure that unhealthy foods are not commercially promoted to children including adolescents in settings where children gather (e.g. preschools, schools, sport and cultural events). |
|  | PROMO5 Effective policies are implemented by the government to ensure that unhealthy foods are not commercially promoted to children, including adolescents on food packages |
| Domain 4 – Food prices    Food pricing policies (e.g., taxes and subsidies) are aligned with health outcomes by  helping to make the healthy eating choices the easier, cheaper choices. | PRICES1 Taxes or levies on healthy foods are minimized to encourage healthy food choices (e.g. low or no sales tax, excise, value-added or import duties on fruit and vegetables). |
|  | PRICES2 Taxes or levies on unhealthy foods (e.g. sugar-sweetened beverages, foods high in nutrients of concern) are in place and increase the retail prices of these foods by at least 10% to discourage unhealthy food choices, and these taxes are reinvested to improve population health. |
|  | PRICES3 The intent of existing subsidies on foods, including infrastructure funding support (e.g. research and development, supporting markets or transport systems), is to favor healthy rather than unhealthy foods. |
|  | PRICES4 The government ensures that food-related income support programs are for healthy foods. |
| Domain 5 – Food provision    The government ensures that there are healthy food service policies implemented in government-funded settings to ensure that food provision encourages healthy food choices, and the government actively encourages and supports private companies to implement similar. | PROV1 The government ensures that there are clear, consistent policies (including nutrition standards) implemented in schools and early childhood education services for food service activities (canteens, food at events, fundraising, promotions, vending machines etc.) to provide and promote healthy food choices. |
|  | PROV2 The government ensures that there are clear, consistent policies in other public sector settings for food service activities (canteens, food at events, fundraising, promotions, vending machines, etc.) to provide and promote healthy food choices. |
|  | PROV3 The government ensures that there are clear, consistent public procurement standards in public sector settings for food service activities to provide and promote healthy food choices. |
|  | PROV4 The Government ensures that there are good support and training systems to help schools and other public sector organizations and their caterers meet the healthy food service policies and guidelines |
|  | PROV5 The Government actively encourages and supports private companies to provide and promote healthy foods and meals in their workplaces. |
| Domain 6 – Food in retail  The government has the power to implement policies and programs to support the availability of healthy foods and limit the availability of unhealthy foods in communities (outlet density and locations) and in-store (product placement). | RETAIL1 Zoning laws and policies are implemented to place limits on the density or placement of quick serve restaurants or other outlets selling mainly unhealthy foods in communities and/or access to these outlets (e.g. opening hours). |
|  | RETAIL2 Zoning laws and policies are implemented to encourage the availability of outlets selling fresh fruit and vegetables and/or access to these outlets (e.g. opening hours, frequency i.e. for markets). |
|  | RETAIL3 The Government ensures existing support systems are in place to encourage food stores to promote the in-store availability of healthy foods and to limit the in-store availability of unhealthy foods. |
|  | RETAIL4 The government ensures existing support systems are in place to encourage the promotion and availability of healthy foods in food service outlets and to discourage the promotion and availability of unhealthy foods in food service outlets. |

### Classification of policy cycle stages

We used the policy cycle as a theoretical framework in this study which was based on the chapter Theories of the Policy Cycle by Jann and Wegrich (2007) in the book Handbook of Public Policy Analysis (8). The described policy stages are agenda-setting, policy formulation, decision-making, implementation, and evaluation. Agenda-setting is explained as problem recognition and issue selection. The problem must first be recognized as a problem that needs policy. After that, the problem needs to be of such urgency that it is set on the ‘agenda’ of the politicians. Policy formulation is the second stage, where one or more possible policies for the problem are suggested. Decision-making follows, where the final adoption of the policy takes place. Then the policy gets implemented by the responsible institutions and organizations: the stage of implementation. The final stage is the evaluation of the policy. The policy is evaluated by the intended outcomes and possible unintended impacts.

Examples from newspaper articles:

Policy formulation:

- Consumer proposes possible policy
- The policy is mentioned in an election program
- The government is investigating if a policy is feasible
- Formulating how the policy will take shape

Decision-making:

- The decision to implement a policy
- The decision about a part of the policy

Decision-making – negative:

- The decision to not implement a policy

Implementation:

- About the implementation process in the work field

Evaluation:

- Is the goal reached?
- What are the effects of the policy?
- Opinion about an implemented policy

### Classification of actors

Based on Hilton et al., (2019) and discussions amongst the research team, we made a classification of actor groups (9). These actors included academics, consumers, policymakers, actors working in public health/ environmental organizations and actors working in the food industry. The category ‘academics’ encompassed members of universities, hospitals or scientific departments. The category ‘consumers’ included (representatives of) Dutch citizens, who could also be the author of the newspaper article if not self-identifying as another actor category. The category ‘policymakers’ represented governmental organizations, members of the States General, local politicians and municipalities. The category ‘actors working in public health/environmental organizations’ encompassed organizations propagating public or environmental health, and non-governmental organizations. The category ‘actors working in the food industry’ included food manufacturers, retailers and federations. If no information regarding actor was available, we coded this as ‘unknown’. Examples of actor groups can be found in Table 3.

*Supplementary Table 3: Examples of actor groups identified in Dutch newspapers:*

| Category | Identified in newspapers |
| --- | --- |
| Academics | Professors (and other academic titles), Wetenschappelijke Raad voor het Regeringsbeleid (WRR), Physicians, Scientific agencies of political parties (think tanks), Nutritionists, University, Medical center, Dieticians, Scientists, Health Council, Hospital employees. |
| Consumers | Respondents survey, Article writer, Consumers Union. |
| Public health/environmental organizations | Foodwatch, Rijksinstituut voor Volksgezondheid en Milieu (RiVM), Gemeentelijke gezondheidsdienst (GGD), Voedingscentrum, Jongeren op Gezond Gewicht (JOGG), Raad van volksgezondheid en samenleving (RVS), Diabetes fonds, Cardea jeugdzorg, Wakker dier, Hartstichting, Raad Leefomgeving en Infrastructuur (RLI), Milieu Defensie, True Animal Protein Price (TAPP) coalitie, Varkens in Nood, ‘Even geen vlees’, Nierstichting. |
| Policymakers | Ministers, State Secretaries, Aldermen, Political Parties, The Government, Municipalities (Board), Cities, Ministry, Central Planning Bureau (CPB). |
| Food industry | Federatie Nederlandse Levensmiddelen Industrie (FNLI), Unilever, Koninklijke Horeca Nederland (KHN), Local entrepreneur, ‘Ik kiest bewust’, Supermarkets, Dutch Association of Dieticians, Vegetable manufacturer, HAK, Centrum Bureau Levensmiddelenhandel (CBL), The Industry, Vereniging van erkende Reclame Advies Bureau (VEA), Fruitfarmers, The Meat sector, Suiker Unie, Frisdranken, Waters en Sappen (FWS), Coca-Cola. |

### Classification of themes of argumentation

In line with Rowbotham et al., (2019) we classified the themes of argumentation of the actor into health, societal, economic, practical and cultural/ideological (10). The health theme included arguments that focused on general health consequences or specific arguments regarding the impact of the food policy on health. The societal theme encompassed arguments regarding consequences for vulnerable groups within the society, such as children or elderly. The economic theme included general or specific arguments of economic consequences for instance raising revenue. The practical theme included arguments about practical enablers or barriers such as feasibility and acceptability. The cultural/ideological theme encompassed arguments that focused on beliefs, norms, values and rights of individuals/organizations/society. This also included political beliefs regarding the involvement and responsibility of individuals/governments. If there was no clear argumentation, we categorized this as no argumentation.

## Data analysis

### Pilot arguments

Based on Hilton et al., (2019); Rowbotham et al., (2019) and discussions amongst the research team, we set up pilot arguments for each theme and viewpoint (Table 4) (9,10).

*Supplementary Table 4: Arguments for each theme and viewpoint regarding food policy issues derived from Hilton et al., (2019); Rowbotham et al., (2019).*

| Themes of arguments | Supportive | Opposed |
| --- | --- | --- |
| Health | Link between dietary intake/component and health  f.e. sugar, fat, and illness, non-communicable diseases | Minimizes the link between dietary intake/component and health |
|  | (Evidence that) Policy will have a positive impact on health (effective)  f.e. reducing morbidity/mortality  (but also reformulation which leads to a decrease) | Lack of evidence that policy will have a positive impact on health (not effective)  f.e. little real-life evidence, people will switch to alternatives |
| Societal | Policy protects vulnerable people  f.e. children, elderly, low-income, inequalities | Policy will harm vulnerable people  f.e. low-income, inequalities |
| Economic | Policy will have a positive economic impact  f.e. raises revenue | Policy will have a negative economic impact  f.e. on small businesses, cost jobs |
|  | Policy will not harm business  f.e. comparison with other options |  |
| Practical | Policy is effective (specific about the policy)  f.e. referring to real-life evaluations | Policy is not effective (specific about the policy)  f.e. flaws in the policy |
|  | Support towards policy  f.e. public or political will | Support is lacking towards policy  f.e. public or political will |
|  | Policy is appropriate  f.e. there are enough alternatives for SSB | Policy is not feasible  f.e. difficulties in implementation |
|  |  | Policy is not appropriate  f.e. other measures would be more effective, sugar cannot be regulated, obesity is too complex, |
|  |  | Policy is unnecessary  f.e. self-regulation industry |
| Cultural/Ideological | More upstream (policy) is needed to overcome problem  f.e. marketing ban | Government should not interfere  f.e. ‘verzorgingsstaat’ |
|  | Government is responsible to support people  f.e. in making right decisions | Individual is responsible  f.e. for own behavior |
|  | Sends a clear message  f.e. sugar is bad for you | Threat to rights  f.e. no free choice |
|  |  | Motivation  f.e. government has different motivations for policy |

###

### Reflexivity

NMSD, a white female, was the primary assessor in this study regarding the actors’ viewpoints and arguments, which was her thesis in the Master of Health Sciences, Nutrition and Health. Before this, she finished her bachelor’s degree in Health and Life Sciences. She worked for several months as a student assistant in methodological courses but has no formal work experience in the research field. NS, a white female, was the primary assessor in this study regarding the identification of the policy cycle stages, which was her thesis in the Master of Health Sciences, Health Policy. She has no formal work experience in the research field. SCD and JDM, both white females, were the supervisors during the entire study. SCD is an assistant professor at the Department of Public and Occupational Health at the Amsterdam University Medical Centers. JDM is an assistant professor at the Department of Epidemiology and Data Science at the Amsterdam Public Health Research Institute. They have ample experience with qualitative studies in the domain of public health nutrition. The decisions that were made in this study were based on literature and discussions with supervisors. We conducted an interpretive content analysis which we believed was the best approach for the aim of this study. However, due to this methodological approach, interpretation of the data is needed. The experiences and perspectives of all assessors may potentially have influenced the interpretation and analysis of the data.

# **Extended results**

## Search and selection process

The literature search generated a total of 4632 references (Figure 1 in manuscript). After removing duplicates, 4347 references remained. In total, 896 articles satisfied the inclusion criteria. Articles were removed because no compulsory national-level Dutch food policy was covered or articles were not newspaper articles (e.g., puzzles, tv-program explanations and recipes.).

## Overview of volume and content of food policies in Dutch newspapers

In the 896 included newspaper articles, food policies were mentioned 1464 times. Regarding the content of newspaper articles, the majority of the newspaper articles reported on food price policies. Other food domains were mentioned less often (Table 5). All newspapers had approximately equal coverage of the different food policy domains (Figure 1).

*Supplementary Table 5: Distribution of volume and content of food policies mentioned in Dutch newspapers.*

|  | | Food prices | Food labeling | Food promotion | Food in retail | Food provision | Food composition | Total (%) |
| --- | --- | --- | --- | --- | --- | --- | --- | --- |
| Newspape r | AD | 125 | 10 | 13 | 9 | 6 | 3 | 166 (11,3) |
|  | ND | 91 | 9 | 9 | 9 | 5 | 4 | 127 (8,7) |
|  | NRC | 201 | 23 | 20 | 22 | 13 | 5 | 284 (19,4) |
|  | Reformato- risch Dagblad | 65 | 15 | 5 | 3 | 2 | 5 | 95 (6,4) |
|  | Telegraaf | 192 | 24 | 12 | 23 | 5 | 10 | 266 (18,1) |
|  | Trouw | 193 | 12 | 9 | 15 | 12 | 9 | 250 (17,1) |
|  | Volkskrant | 211 | 17 | 16 | 9 | 12 | 11 | 276 (18,9) |
| Total (%) | | 1078 (73,6) | 110 (7,5) | 84 (5,7) | 90 (6,2) | 55 (3,8) | 47 (3,2) | 1464 (100) |

*Supplementary Figure 1: Distribution of volume and content of food policies mentioned per Dutch newspaper.*

## Overview of policy cycle stages

Newspaper articles mainly described food policies in the stage of policy formulation (Table 6). Other stages of the policy cycle were mentioned less often.

*Supplementary Table 6:* *Overview of identified policy cycle stages of food policies in Dutch newspapers.*

|  | Policy formulation | Decision making | Evaluation | Implementation |
| --- | --- | --- | --- | --- |
| 2000 | 0 | 0 | 1 | 0 |
| 2001 | 2 | 0 | 0 | 0 |
| 2002 | 0 | 0 | 0 | 0 |
| 2003 | 8 | 0 | 0 | 0 |
| 2004 | 25 | 0 | 1 | 0 |
| 2005 | 42 | 1 | 0 | 0 |
| 2006 | 9 | 0 | 0 | 0 |
| 2007 | 57 | 1 | 0 | 0 |
| 2008 | 28 | 1 | 0 | 0 |
| 2009 | 48 | 0 | 0 | 0 |
| 2010 | 50 | 0 | 1 | 0 |
| 2011 | 72 | 0 | 0 | 0 |
| 2012 | 70 | 0 | 0 | 0 |
| 2013 | 34 | 0 | 3 | 0 |
| 2014 | 23 | 0 | 0 | 0 |
| 2015 | 23 | 0 | 0 | 1 |
| 2016 | 54 | 2 | 0 | 0 |
| 2017 | 48 | 0 | 0 | 0 |
| 2018 | 123 | 9 | 3 | 0 |
| 2019 | 89 | 21 | 3 | 0 |
| 2020 | 69 | 5 | 0 | 0 |
| 2021 | 252 | 5 | 0 | 0 |
| 2022 | 253 | 26 | 0 | 1 |
| Total (N%) | 1379 (94,2%) | 71 (4,9%) | 12 (0,8%) | 2 (0,1%) |

## Overview of viewpoint actors and arguments

In the subsample (40% of the newspaper articles regarding food composition, food labeling, food promotion, food provision and food in retail, and 20% regarding food prices), a total of 1195 unique arguments were identified (Table 7). Consumers (represented in 34,6% of the newspaper articles), academics (26,2%), policymakers (21,6%) and public health/environmental professionals (12,9%) were mainly supportive of the food policies from all food domains (Table 8). Only actors from the food industry (4,8%) were mostly opposed to the food policies. In general, a neutral viewpoint was rarely identified for all actors. Details on viewpoint and used arguments per food domain can be found in Table 9.

*Supplementary Table 7: Actors’ arguments identified in Dutch newspaper articles regarding food policies.*

| Viewpoint | Academics | Consumers | Public health/ environmental organizations | Policymakers | Food industry | Total |
| --- | --- | --- | --- | --- | --- | --- |
| Supportive arguments | 265 | 273 | 140 | 153 | 14 | 845 |
| Opposing arguments | 47 | 132 | 13 | 91 | 42 | 325 |
| Neutral | 1 | 8 | 1 | 14 | 1 | 24 |
| Total | 313 | 413 | 154 | 258 | 57 | 1195 |

*Supplementary Table 8: Distribution of prevalence (%) of actors in Dutch newspaper articles regarding food policies.*

|  | Total | Supportive | Opposing | Neutral |
| --- | --- | --- | --- | --- |
| Consumers | 34,6 | 66,1 | 32 | 1,9 |
| Academics | 26,2 | 84,7 | 15 | 0,3 |
| Policymakers | 21,6 | 59,3 | 35,3 | 5,4 |
| PH/Environ. | 12,9 | 90,9 | 8,4 | 0,7 |
| Food industry | 4,8 | 24,6 | 73,7 | 1,7 |

*Supplementary Table 9: Detailed viewpoint and used arguments per food domain by different actors in Dutch newspaper articles regarding food policies.*

| Actor: | Food domains: | Main viewpoint: |
| --- | --- | --- |
| Academics | Food composition | Supportive - ideological |
|  | Food labeling | Supportive - human health |
|  | Food promotion | Supportive – ideological (also many human health) |
|  | Food prices | Supportive – ideological (also many human health) |
|  | Food provision | Supportive - ideological |
|  | Food in retail | Supportive - ideological |
| Consumers | Food composition | Supportive - ideological |
|  | Food labeling | Supportive - human health |
|  | Food promotion | Supportive - human health |
|  | Food prices | Supportive - ideological |
|  | Food provision | Supportive – human health & ideological |
|  | Food in retail | Supportive - ideological |
| Policymakers | Food composition | Even* |
|  | Food labeling | Supportive - human health |
|  | Food promotion | Even* |
|  | Food prices | Supportive - human health & planetary health |
|  | Food provision | Supportive - human health |
|  | Food in retail | Supportive - human health |
| PH / environmental organizations | Food composition | Supportive - human health |
|  | Food labeling | Supportive - ideological |
|  | Food promotion | Supportive - ideological |
|  | Food prices | Supportive - human health |
|  | Food provision | Even* |
|  | Food in retail | Supportive - ideological |
| Food industry | Food composition | Opposing - practical |
|  | Food labeling | Supportive - human health |
|  | Food promotion | Opposing - human health & practical |
|  | Food prices | Opposing - human health |
|  | Food provision | N.A. |

*An even main viewpoint meant that there were as many supportive as opposed arguments.

# SRQR Checklist

|  | Reporting Item | Page Number |
| --- | --- | --- |
| Title & Abstract |  |  |
|  | Title: Concise description of the nature and topic of the study identifying the study as qualitative or indicating the approach (e.g. ethnography, grounded theory) or data collection methods (e.g. interview, focus group) is recommended | 1 |
|  | Abstract:  Summary of the key elements of the study using the abstract format of the intended publication; typically includes background, purpose, methods, results and conclusions | 2 |
| Introduction |  |  |
|  | Problem formulation: Description and significance of the problem / phenomenon studied: review of relevant theory and empirical work; problem statement | 3,4 |
|  | Purpose or research question: Purpose of the study and specific objectives or questions | 4,5 |
| Methods |  |  |
|  | Qualitative approach and research paradigm: Qualitative approach (e.g. ethnography, grounded theory, case study, phenomenolgy, narrative research) and guiding theory if appropriate; identifying the research paradigm (e.g. postpositivist, constructivist / interpretivist) is also recommended; rationale. The rationale should briefly discuss the justification for choosing that theory, approach, method or technique rather than other options available; the assumptions and limitations implicit in those choices and how those choices influence study conclusions and transferability. As appropriate the rationale for several items might be discussed together. | 5, S2 |
|  | Researcher characteristics and reflexivity:  Researchers' characteristics that may influence the research, including personal attributes, qualifications / experience, relationship with participants, assumptions and / or presuppositions; potential or actual interaction between researchers' characteristics and the research questions, approach, methods, results and / or transferability | S13 |
|  | Context:  Setting / site and salient contextual factors; rationale | 5-7 |
|  | Sampling strategy:  How and why research participants, documents, or events were selected; criteria for deciding when no further sampling was necessary (e.g. sampling saturation); rationale | 5-7 |
|  | Ethical issues pertaining to human subjects:  Documentation of approval by an appropriate ethics review board and participant consent, or explanation for lack thereof; other confidentiality and data security issues | 18 |
|  | Data collection methods: Types of data collected; details of data collection procedures including (as appropriate) start and stop dates of data collection and analysis, iterative process, triangulation of sources / methods, and modification of procedures in response to evolving study findings; rationale | 5-7, S2-10 |
|  | Data collection instruments and technologies: Description of instruments (e.g. interview guides, questionnaires) and devices (e.g. audio recorders) used for data collection; if / how the instruments(s) changed over the course of the study | 5-7, S2-3 |
|  | Units of study: Number and relevant characteristics of participants, documents, or events included in the study; level of participation (could be reported in results) | 8, S14 |
|  | Data processing:  Methods for processing data prior to and during analysis, including transcription, data entry, data management and security, verification of data integrity, data coding, and anonymisation / deidentification of excerpts | 4-6, S2-10 |
|  | Data analysis: Process by which inferences, themes, etc. were identified and developed, including the researchers involved in data analysis; usually references a specific paradigm or approach; rationale | 7-8, S2-13 |
|  | Techniques to enhance trustworthiness: Techniques to enhance trustworthiness and credibility of data analysis (e.g. member checking, audit trail, triangulation); rationale | 7-8 |
| Results/findings |  |  |
|  | Syntheses and interpretation: Main findings (e.g. interpretations, inferences, and themes); might include development of a theory or model, or integration with prior research or theory | 8-13 |
|  | Links to empirical data: Evidence (e.g. quotes, field notes, text excerpts, photographs) to substantiate analytic findings | 8-13, S14-19 |
| Discussion |  |  |
|  | Integration with prior work, implications, transferability and contribution(s) to the field: Short summary of main findings; explanation of how findings and conclusions connect to, support, elaborate on, or challenge conclusions of earlier scholarship; discussion of scope of application / generalizability; identification of unique contributions(s) to scholarship in a discipline or field | 13-17 |
|  | Limitations: Trustworthiness and limitations of findings | 16 |
| Other |  |  |
|  | Conflicts of interest: Potential sources of influence of perceived influence on study conduct and conclusions; how these were managed | 18 |
|  | Funding: Sources of funding and other support; role of funders in data collection, interpretation and reporting | 18 |

# References

1. Nationaal Onderzoek Multimedia 2023. Available from: <https://www.nommedia.nl/>.
2. Djojosoeparto SK, Kamphuis CBM, Vandevijvere S, Poelman MP. How can National Government Policies Improve Food Environments in the Netherlands? International Journal of Public Health. 2022;67:1604115.
3. Swinburn B, Sacks G, Vandevijvere S, Kumanyika S, Lobstein T, Neal B, et al. INFORMAS (International Network for Food and Obesity/non-communicable diseases Research, Monitoring and Action Support): overview and key principles. Obes Rev. 2013;14 Suppl 1:1-12.
4. Kelishadi R, Farajian S. The protective effects of breastfeeding on chronic non-communicable diseases in adulthood: A review of evidence. Advanced Biomedical Research. 2014;3:3.
5. Rowbotham S, McKinnon M, Marks L, Hawe P. Research on media framing of public policies to prevent chronic disease: A narrative synthesis. Social Science & Medicine. 2019;237.
6. Hawkins B, Holden C. Framing the alcohol policy debate: industry actors and the regulation of the UK beverage alcohol market. Critical Policy Studies. 2013;7(1):53-71.
7. Fogarty AS, Chapman S. Advocates, interest groups and Australian news coverage of alcohol advertising restrictions: content and framing analysis. BMC Public Health. 2012;12(1):727.
8. Jann, W., & Wegrich, K. (2007). Theories of the Policy Cycle. In F. Fischer, G. J. Müller, & M. S. Sidney (Eds.), Handbook of Public Policy Analysis (1st ed., p. 20). Routledge.
9. Hilton S, Buckton CH, Patterson C, Katikireddi SV, Lloyd-Williams F, Hyseni L, et al. Following in the footsteps of tobacco and alcohol? Stakeholder discourse in UK newspaper coverage of the Soft Drinks Industry Levy. Public Health Nutrition. 2019;22(12):2317-28.
10. Rowbotham S, McKinnon M, Marks L, Hawe P. Research on media framing of public policies to prevent chronic disease: A narrative synthesis. Social Science & Medicine. 2019;237.
