# Supplementary material for: Unveiling viewpoints on national food environment policies in the Dutch newspaper discourse: an interpretative media content analysis
Source: Int J Behav Nutr Phys Act. 2024 Jul 24;21:80. doi: 10.1186/s12966-024-01625-3 (PMC11267762; doi:10.1186/s12966-024-01625-3)
Supplement: Supplementary file 2 — Supplementary Material 2. [file 12966_2024_1625_MOESM2_ESM.docx]

Amsterdam University Medical Centers	Department of Public and Occupational Health
De Boelelaan 1089a
1081HV Amsterdam
The Netherlands

Email: [j.mackenbach@amsterdamumc.nl](mailto:j.mackenbach@amsterdamumc.nl)

July 1 2024

Prof. R. Rosenkranz
Prof. M. Hingle,
Editors-in-Chief
International Journal of Behavioral Nutrition and Physical Activity

Concerning: Manuscript revision (IJBN-D-24-00176)

Dear Prof. Rosenkranz, Hingle,

Please find enclosed our revised manuscript, “Unveiling viewpoints on national food policies in the Dutch newspaper discourse: an interpretative media content analysis”. We have addressed the remaining editorial comments.

We look forward to hearing from you in due course.

Yours Sincerely,

Joreintje D. Mackenbach (On behalf of the co-authors)

Editor comments:

1. "Conclusion" subheading in the main body should be replaced by "Conclusions"

This has been changed.

2. The competing interests declaration should include "Author AB is a member of the Editorial Board of International Journal of Behavioral Nutrition and Physical Activity. AB was not involved in the journal’s peer review process of, or decisions related to, this manuscript."

This has been changed.

3. Funder's role is missing.

This has been added.
